# Interdisciplinary and Collaborative Training in Neuroscience: Insights from the Human Brain Project Education Programme

**DOI:** 10.1007/s12021-024-09682-6

**Published:** 2024-11-06

**Authors:** Alice Geminiani, Judith Kathrein, Alper Yegenoglu, Franziska Vogel, Marcelo Armendariz, Ziv Ben-Zion, Petrut Antoniu Bogdan, Joana Covelo, Marissa Diaz Pier, Karin Grasenick, Vitali Karasenko, Wouter Klijn, Tina Kokan, Carmen Alina Lupascu, Anna Lührs, Tara Mahfoud, Taylan Özden, Jens Egholm Pedersen, Luca Peres, Ingrid Reiten, Nikola Simidjievski, Inga Ulnicane, Michiel van der Vlag, Lyuba Zehl, Alois Saria, Sandra Diaz-Pier, Johannes Passecker

**Affiliations:** 1 Department of Brain and Behavioural Sciences, University of Pavia, via Forlanini, 6, Pavia, 27100 Italy; 2 Present Address: Champalimaud Foundation, Avenida Brasília, Lisbon, 1400-038 Portugal; 3 Institute of Neurobiochemistry, Medical University Innsbruck, Innrain 80-82, Innsbruck, 6020 Tyrol Austria; 4 Simulation and Data Lab Neuroscience, Jülich Supercomputing Centre, Forschungszentrum Jülich GmbH, Wilhelm-Johnen-Str, Jülich, 52425 NRW Germany; 5 Boston Children’s Hospital, Harvard Medical School, 300 Longwood Ave, Boston, Massachusetts 02115 USA; 6 Departments of Comparative Medicine and Psychiatry, Yale University School of Medicine, 333 Cedar St, New Haven, CT 06510 USA; 7 United States Department of Veterans Affairs National Center for PTSD, Clinical Neuroscience Division, VA Connecticut Healthcare System, 950 Campbell Ave, West Haven, CT 06516 USA; 8 Innatera Nanosystems, Patrijsweg 20, Rijswijk, 2289 EX Netherlands; 9 Institut d’Investigacions Biomédiques August Pi i Sunyer (IDIBAPS), C/ del Rosselló 149, Barcelona, 08036 Spain; 10 CONVELOP cooperative knowledge design gmbh, Kaiserfeldgasse 7, Graz, 8010 Styria Austria; 11 FAST, Cadence Design Systems, 1 Penrose, Cork, T23KW81 Cork Ireland; 12 Experimental Psychiatry Unit, Department of Psychiatry 1, Medical University Innsbruck, Innrain 80-82, Innsbruck, 6020 Tyrol Austria; 13 Institute of Biophysics, National Research Center, via Ugo la Malfa n. 153, Palermo, 90146 Italy; 14 Department of Sociology, University of Essex, Wivenhoe Park, Colchester, CO4 3SQ UK; 15 Department of Computer Science, Technical University of Darmstadt, Hochschulstr. 10, 64289 Hesse Darmstadt, Germany; 16 Computational Science and Technology, KTH Royal Institute of Technology, Lindstedtsvägen 5, Stockholm, 11428 Stockholm Sweden; 17 Department of Computer Science, The University of Manchester, Oxford Rd, Manchester, M13 9PL UK; 18 Neural Systems Laboratory, Department of Molecular Medicine, Institute of Basic Medical Sciences, University of Oslo, Sognsvannsveien 9, Oslo, 0372 Oslo Norway; 19 Department of Oncology, University of Cambridge, B 197, Hutchison-MRC Research Centre, Cambridge Biomedical Campus, Hills Road, Cambridge, CB2 0XZ UK; 20 University of Birmingham, Edgbaston, Birmingham, B15 2TT UK; 21 EBRAINS AISBL, Chau. de la Hulpe, Watermael-Boitsfort, Brussels, 1170 Belgium; 22 Office for (Inter-)national Coordination and Networking, Jülich Supercomputing Centre, Forschungszentrum Jülich, Wilhelm-Johnen-Str, 52425 NRW Jülich, Germany

**Keywords:** Neuroscience training, Education, Interdisciplinarity, Digital research infrastructure, Human brain project

## Abstract

Neuroscience education is challenged by rapidly evolving technology and the development of interdisciplinary approaches for brain research. The Human Brain Project (HBP) Education Programme aimed to address the need for interdisciplinary expertise in brain research by equipping a new generation of researchers with skills across neuroscience, medicine, and information technology. Over its ten year duration, the programme engaged over 1,300 experts and attracted more than 5,500 participants from various scientific disciplines in its blended learning curriculum, specialised schools and workshops, and events fostering dialogue among early-career researchers. Key principles of the programme’s approach included fostering interdisciplinarity, adaptability to the evolving research landscape and infrastructure, and a collaborative environment with a focus on empowering early-career researchers. Following the programme’s conclusion, we provide here an analysis and in-depth view across a diverse range of educational formats and events. Our results show that the Education Programme achieved success in its wide geographic reach, the diversity of participants, and the establishment of transversal collaborations. Building on these experiences and achievements, we describe how leveraging digital tools and platforms provides accessible and highly specialised training, which can enhance existing education programmes for the next generation of brain researchers working in decentralised European collaborative spaces. Finally, we present the lessons learnt so that similar initiatives may improve upon our experience and incorporate our suggestions into their own programme.

**Supplementary Information:**

The online version contains supplementary material available at 10.1007/s12021-024-09682-6.

**Fig. 1:**
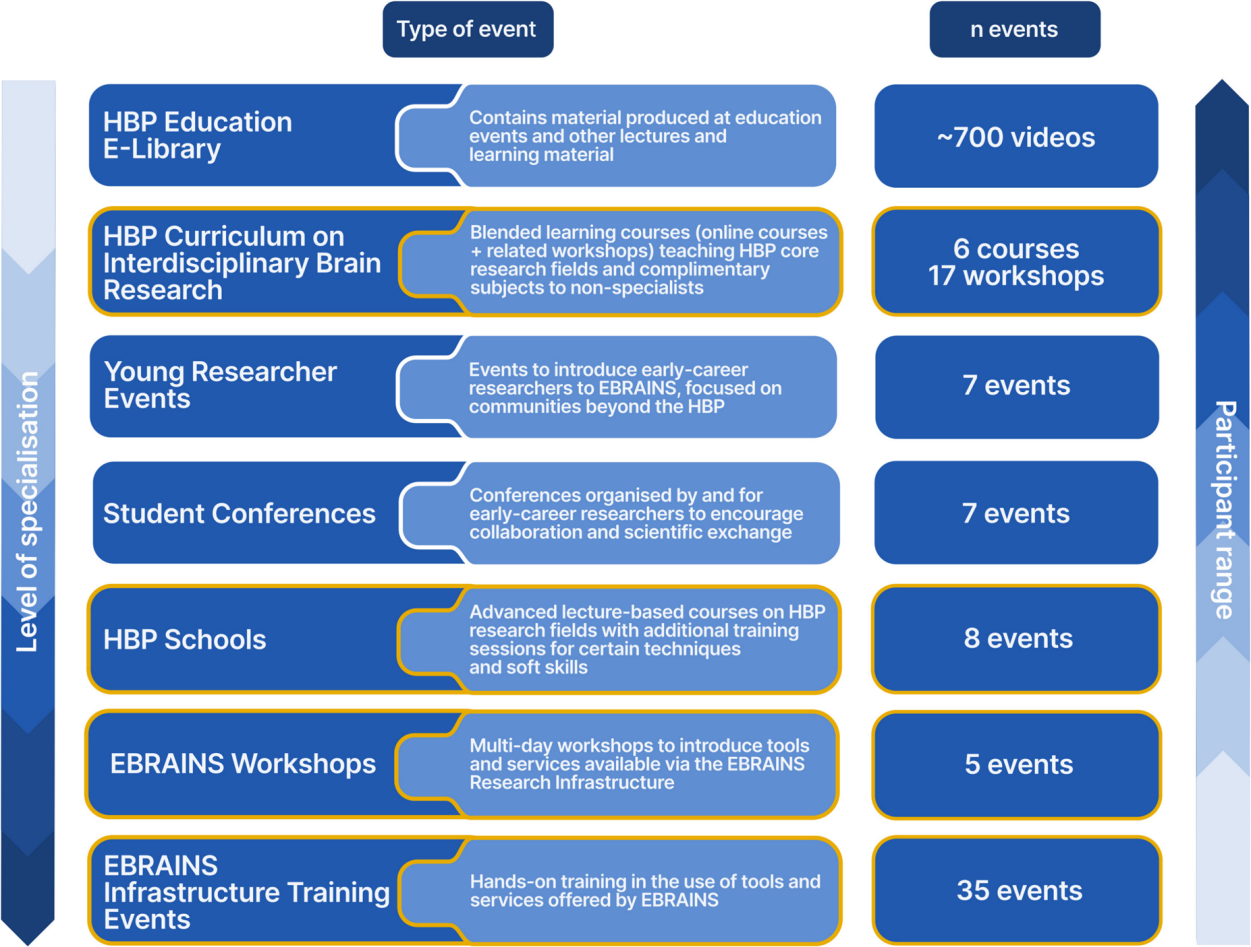
Overview of the main HBP Education Programme event formats. The education formats covered increasing levels of specialisation. While specialised events targeted a more narrow range of participants, high-level formats, such as the HBP Education E-Library, provided learning content to a wide range of participants. Event formats framed yellow were supported via Open Calls for administrative and financial support. The top four formats focusing on increased reach and early-career researchers are discussed in more detail in the following Sections Value of Adaptable Training and Approaches and  HBP Student Events

## Introduction

Achieving a complete understanding of the brain and the way it functions continues to be a challenge. In this investigative pursuit, the fields of neuroscience and engineering have integrated on many levels over the last decades, (Altimus et al., 2020) resulting in a deeply interdisciplinary research field. This synergy has been fueled by rapidly evolving methods and technology that enable collection of large datasets, analysis on an unprecedented scale and large-scale simulations, e.g. high-resolution recording techniques and high-performance computing. Some of these methods were not new as such and were widely used by other communities but not yet applied for neuroscientific research. Neuroengineering, neuroinformatics and computational neuroscience research fields have emerged, but these developments require educational and training efforts to equip both emerging talent and established scientists with the skills and mindsets to remain competitive and at the forefront of discovery (Akil et al., 2016; Litt, 2015; Buhusi et al., 2023). While some universities and institutions offer courses combining computer science and neuroscience (Fox, 2007), there is a scarcity of reports on integrating computational approaches in neuroscience curricula (Latimer et al., 2019) and the traditional mathematics requirements for neuroscience majors are often insufficient for analysing big data sets in contemporary neuroscience research (Hoy, 2021). As a response to the scarcity of interdisciplinary training in traditional education programmes, specific training initiatives have been developed.

At local levels, in the United States the Johns Hopkins’ NeuroEngineering Training Initiative (NETI) and the UCLA’s neuroengineering programme for example provide an interdisciplinary offer combining engineering, mathematics, and neuroscience to address the growing need for computational skills in neuroscience (Davidovics & Colon, 2007; Judy & Tobin, 2003), while other postgraduate programmes in neuroengineering have been launched in Europe and North America, specifically for implantable and wearable neural technologies (Ghannam et al., 2020). On a cross-institutional level, the Advanced School AI (AS-AI)^1^ is an example for an interdisciplinary training programme in neuroengineering with a focus on machine learning and brain modelling.

At a larger scale, the International Neuroinformatics Coordinating Facility (INCF) was established in 2005 to coordinate global neuroinformatics research and resources, with a focus on developing international standards for neuroscience data and models (de Schutter, 2009). Recognising the gap between traditional neuroscience curricula and the computational skills required for big data analysis, the INCF Training and Education Committee developed the *INCF Training Space* (INCF, 2024), an open-access hub of neuroscience educational resources launched in 2019 (George et al., 2022). Computational neuroscience and neuroinformatics training resources and databases are also available from the Allen Institute for Brain Science (Allen Institute, 2024a), together with workshops on specific topics (Allen Institute, 2024b). In Europe, interdisciplinary neuroscience training courses are also offered within the Cajal advanced neuroscience training programme (Cajal Programme, 2024).

In this context, we here discuss the educational effort implemented within the Human Brain Project (HBP). As one of several major international initiatives to investigate the brain, the HBP was launched in 2013 to pioneer an integrated research approach that combines neuroscience, medicine as well as information and communication technology (ICT) to gain a new level of understanding of the brain (Markram et al., 2011). To achieve this, experts capable of working at the intersection of these fields were needed, but scientists with such interdisciplinary knowledge were scarce at the time. To help address this gap, the HBP Education Programme was set up with four major aims:

- Attract and equip talents with essential skills across science, technology, innovation, and responsible research.
- Provide early-career researchers with foundational education in complementary disciplines for interdisciplinary research.
- Offer space for extensive intra- and interdisciplinary dialogue to a new generation of researchers to foster cross-disciplinary collaborations.
- Extend education opportunities to the broader scientific community and the public.

To serve these aims, the HBP Education Programme implemented a framework of education formats, for an interdisciplinary audience with different skill and experience levels (see  Fig. 1). Over the duration of the Education Programme, more than 1,300 experts volunteered to train, teach and create content for more than 5,500 participants from multiple scientific disciplines. This resulted in 700 educational videos and almost 100 training and educational events, in-person and online^2^.

In the following, we discuss how the HBP Education Programme successfully addressed three main challenges, in alignment with the original objectives outlined above: 1) Equipping brain researchers with interdisciplinary knowledge and skills (Section Value of an Interdisciplinary Approach); 2) Adapting neuroscience training programmes to the rapidly changing landscape of digital tools for brain research and education (Section Value of Adaptable Training and Approaches); and 3) Making education pivotal in increasing synergies and collaborations among different fields, career stages and international brain research initiatives (Section Value of a Collaborative Approach). In conclusion, for all these challenges, we highlight the impact the HBP Education Programme had on the HBP and neuroscience research (Section Impact Highlights). Starting from a summary of the lessons learnt, we also provide recommendations for future educational events and activities that could be incorporated into similar large-scale research programmes or implemented on a wider European level (Section Towards Integrated Neuroscience Education).

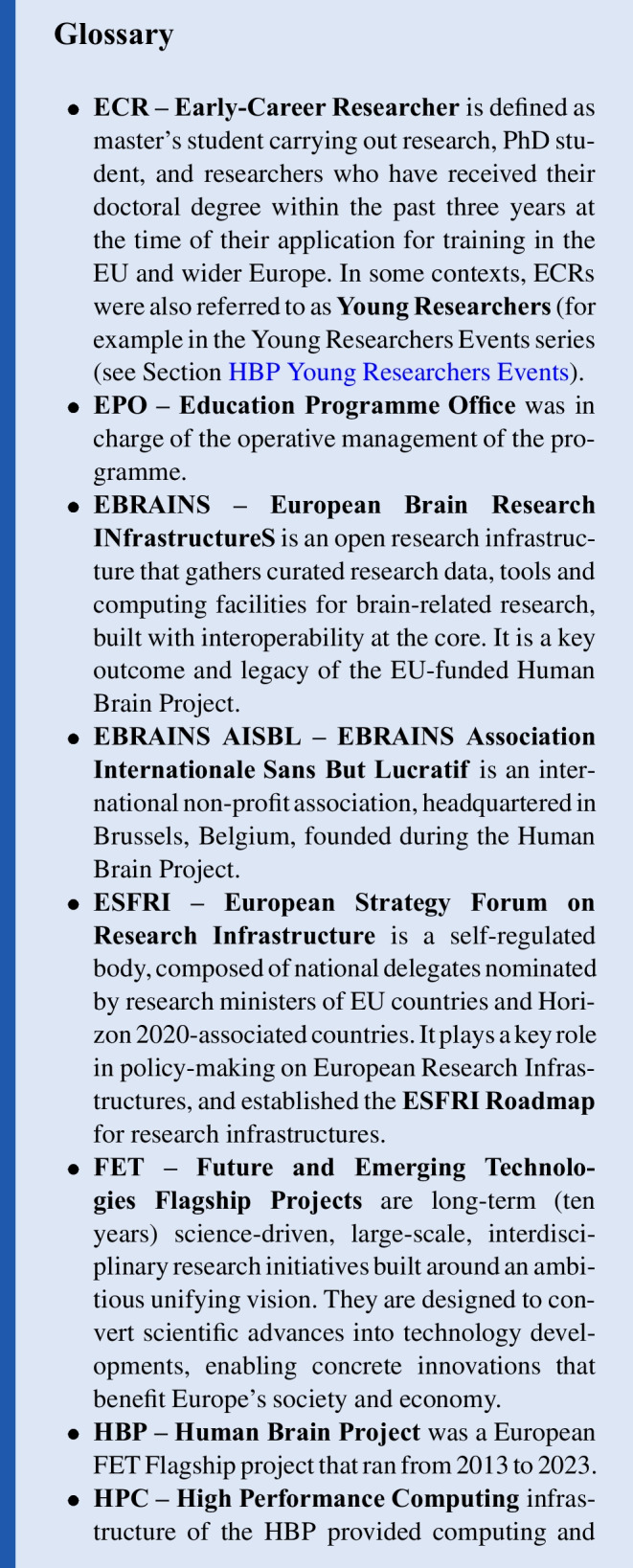

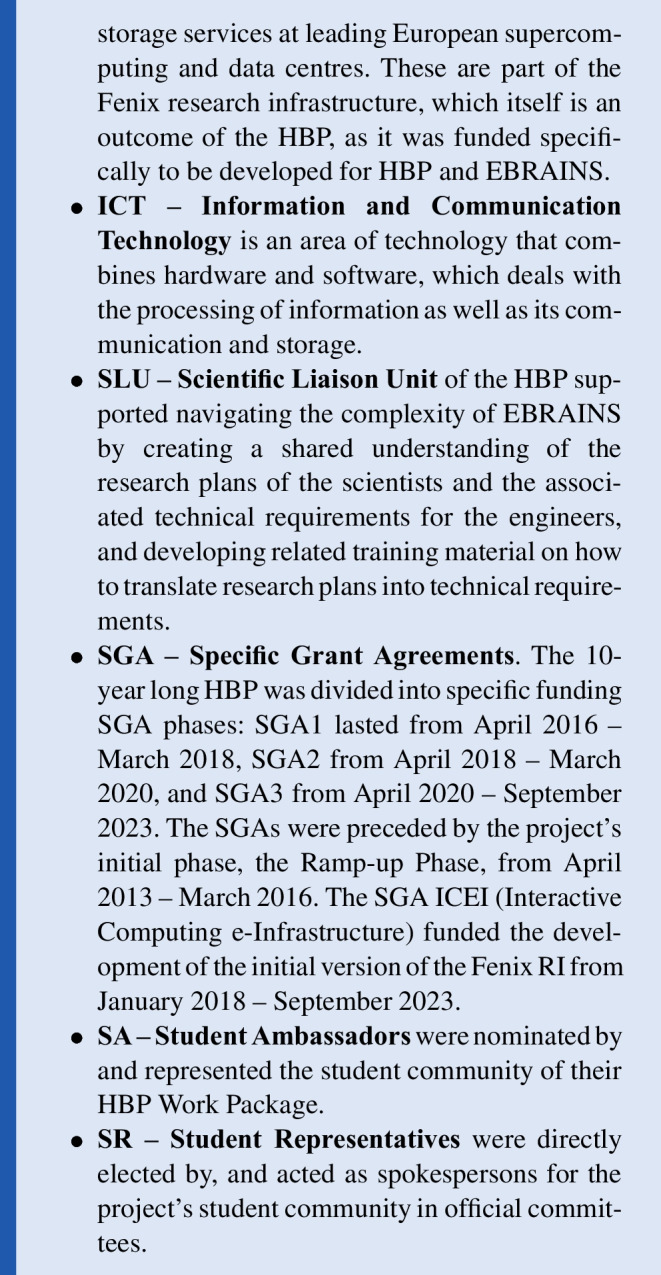





**Fig. 2:**
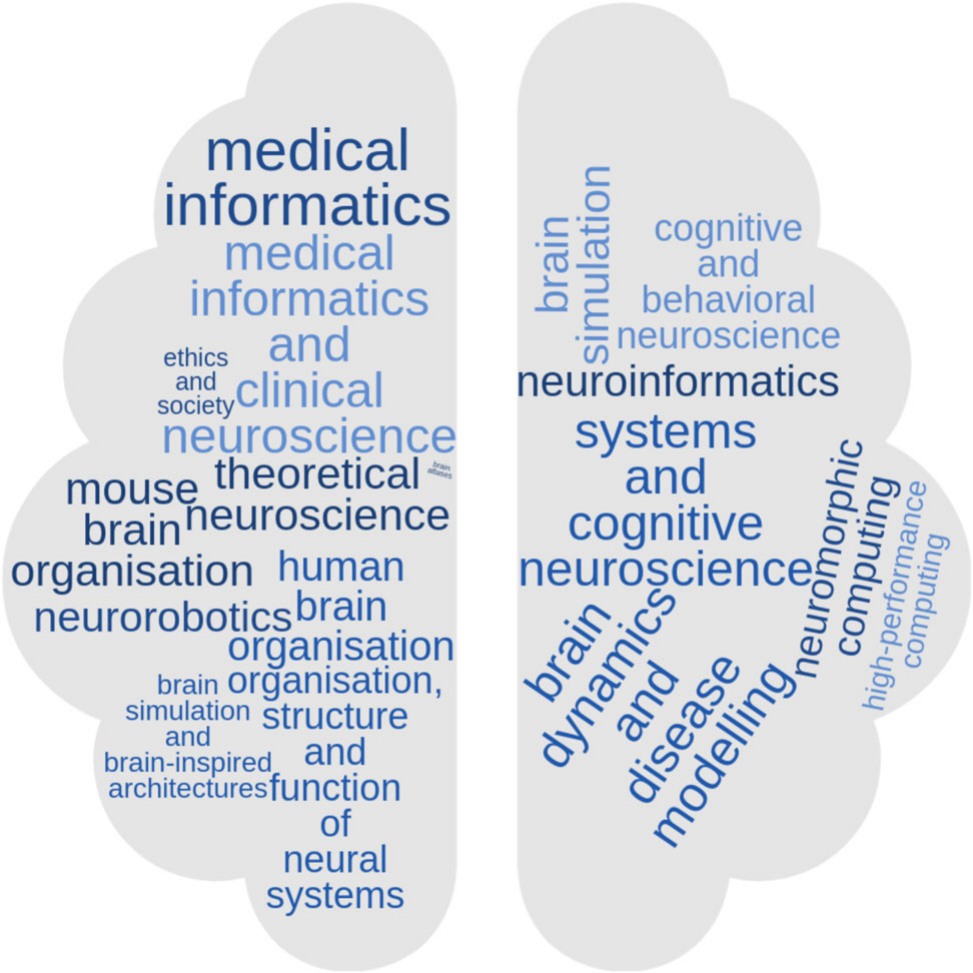
Abstract topics for HBP Student Conferences. The word cloud illustrates topics presented at 6 out of 7 HBP Student Conferences, covering a total of 395 abstract topics. It shows the wide spectrum of disciplines represented in the conferences

## Value of an Interdisciplinary Approach

Recent research efforts have highlighted the importance of interdisciplinarity in brain research (Dyson, 2012; Huang & Luo, 2015). The benefits are two-fold: (1) for neuroscience, to fully exploit novel technologies and neuroinformatics tools to improve data collection, experimental design, and data analysis, and derive theoretical models on brain function; and (2) for engineering and ICT, to develop novel technologies inspired by brain functioning. In this section we will describe how the HBP Education Programme addressed the interdisciplinary nature of the project and aimed at enhancing knowledge transfer, collaboration and trust among disciplines.

**Fig. 3:**
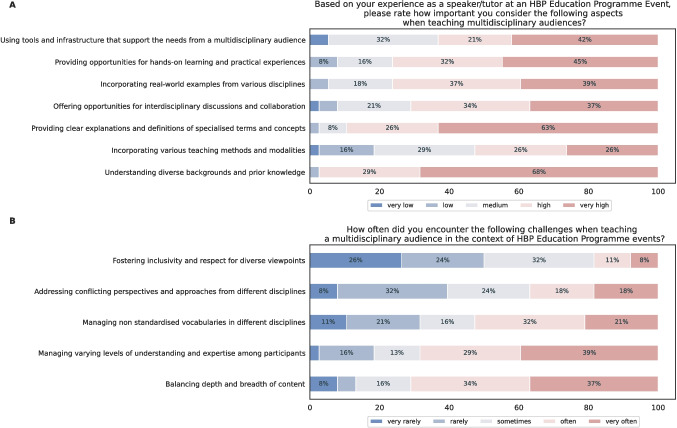
Survey results conducted among speakers and tutors at HBP SGA3 Education events to assess the value and challenges of training an interdisciplinary audience. Entries below $6\%$ are omitted

Leveraging the collective expertise of diverse disciplines is critical. As such, collaborations between, for example, engineers and physicians, statisticians and biologists, or computer scientists and cognitive scientists have become more common. However, these partnerships encounter difficulties, mainly due to differences in terminology, methodologies, and perspective. Bridging these gaps to equip scientists with knowledge and skills that are transversal to disciplines requires interdisciplinary training, common frameworks, and a culture of openness (Gobet, 2018; Grisham et al., 2016). Although brain research concepts were present in university curricula for biology, social sciences, medicine, or computational sciences, at the time of the HBP Education Programme’s conception, there were virtually no interdisciplinary brain science programmes combining these fields and, specifically, the need for more computational and quantitative training had been pointed out for students from life science backgrounds (Goldman & Fee, 2017).

HBP Education events were organised by panels of scientists representing diverse fields and career levels of the HBP. Similarly, events were always open to scientists from all disciplines. Participants ranged from engineers and computer scientists to neurobiologists and medical doctors. As an example, the word cloud in Fig. 2 illustrates the wide spectrum of topics of abstracts submitted to the Student Conferences, showing diverse brain research fields similarly represented. These events aimed at providing ECRs (see Glossary 1) with knowledge and skills from various disciplines independent of their specialisation, and promoting interaction and communication across different fields. As a result, HBP Education training events contributed to acquiring new skills and establishing new collaborations (see Sections Value of Adaptable Training and Approaches and Value of a Collaborative Approach and Supplementary material Section A.1).

The HBP Curriculum on Interdisciplinary Brain Research consisted of four core subject courses (Neurobiology, ICT, Brain Medicine and Cognitive Systems “for non-specialists”), complemented by two courses teaching horizontal topics (’Research Ethics and Societal Impact’, and ’Intellectual Property Rights, Translation and Exploitation of Research’). Since this curriculum was not only directed at students working in the HBP, it was structured as an online course (Section Adaptability of a Blended/Hybrid Educational Approach), with an option for complementary workshops and examinations for receiving ECTS credits.

Interdisciplinary training represents a challenge also for lecturers and teachers. Based on a survey to speakers from the HBP education events, understanding diverse backgrounds and previous knowledge and providing clear explanations and definitions of field-specific concepts and terminology were crucial aspects to be considered when teaching to an interdisciplinary audience, as illustrated in Fig. 3. Indeed, balancing content accessibility and managing varying levels of prior knowledge from the participants were the biggest challenges for the majority of speakers. Using real-world examples, favouring discussion and hands-on experience, exploiting common tools and infrastructure built for interdisciplinary audiences were other important factors to consider. Many speakers considered fostering dialogue and addressing conflicting perspectives among disciplines a very challenging aspect of interdisciplinary teaching. On the other hand, fostering inclusivity and respect for diverse viewpoints was not considered as a significant challenge in the context of interdisciplinary training by the speakers. Whether this reflects a trend in increased respect for inclusion and diversity in interdisciplinary contexts (Baillie et al., 2011) or the need to further increase awareness for speakers could not be identified. A helpful solution could be to provide speakers with student surveys/data prior to events (e.g. diverse backgrounds and knowledge but also sense of inclusion from the participants) so that they can assess all aspects of learning environment prior to lectures.

In conclusion, the HBP Education Programme implemented a set of training activities where organisers, target audience, contents, and teachers represented a wide range of disciplines contributing to brain research. These activities were designed with an interdisciplinary mindset, with the goal of promoting knowledge transfer among scientific fields and in such a way that researchers with a wide range of backgrounds and in different career stages could take advantage of them. Training was mainly focused on neuroinformatics and computer science skills that are required by the rapidly evolving landscape of digital tools for neuroscience, while experimental neuroscience training remains a challenge due to the higher complexity of infrastructure needed (see Lesson 1 in the box 2 in Section Towards Integrated Neuroscience Education).

## Value of Adaptable Training and Approaches

The rapidly evolving landscape of digital tools for brain research and education requires neuroscience education programmes to be flexible and easily adapt to novel neuroinformatics and computational resources available.

### Adaptability to an Evolving Research Infrastructure

Complementary to experimental tools, digital tools are rapidly evolving and widely used in brain research, including online databases for neural data sharing, neuroinformatics for data analysis and software packages for neural circuit modelling and simulation (Neuro Cloud, 2016; Gleeson et al., 2010; Dai et al., 2020a, b). From the outset, the HBP (Amunts et al., 2016) envisioned the design, development, and deployment of a shared ICT and HPC infrastructure (see Glossary 1) to support its research goal. This entailed a collaborative effort to co-design an ICT infrastructure for brain research and to provide specialised ad hoc training on its functionalities and usage to the neuroscience community. Providing continuously updated specialised and hands-on training on the use of these digital tools was essential to increase usability and standardisation, thereby continuously integrating feedback from the end-users (see Lesson 2 in the box 2 in Section Towards Integrated Neuroscience Education).

For example, in March 2016, during SGA1 (see Glossary 1), six initial HBP platforms were launched: neuroinformatics, brain simulation, high-performance analytics and computing, medical informatics, neuromorphic computing and neurorobotics. An adaptive educational programme was required to provide relevant training material alongside the rapid evolution of the platforms.

To make the infrastructure accessible to a wider community and test its potential, several workshops were organised that enabled scientific use cases and covered data, analysis, simulation, and visualisation requirements.

Following the HBP platforms and services consolidation into EBRAINS, voucher programmes (Schneider, 2019) allowed external researchers to participate and learn how to integrate their tools and ideas with the existing hardware and software services. Co-design projects^3^ during SGA2 helped the interconnection of tools, enabling new and complex workflows to be envisioned. This new phase of integration also brought a new wave of training, workshops, and education offers which aimed at helping brain research experts understand the possibilities of exchanging information between tools (e.g. databases, neuroinformatics pipelines for data analysis, modelling and simulation workflows) and maximising the scientific output resulting in these pipelines. With the rise of big data, also questions regarding FAIR principles, reproducible science, good software development practices and cybersecurity started to take a main role in several of the training offerings (Martone, 2003).

In 2021, the EBRAINS RI was selected for the ESFRI Roadmap of European Research Infrastructures (see Glossary 1). This meant that all developers and scientists in the HBP had to focus on enhancing the technology readiness level of their tools, creating thorough documentation and following strict guidelines of technical integration to ensure higher adoption, integration, and standardisation of the infrastructure. The education and training activities developed around the infrastructure became an essential feedback loop to enhance its usability and quality during the last phases of the project.

These processes were supported and partly directed by the Education Programme Office (EPO, see Glossary 1) to implement feedback mechanisms, such as regular participants’ surveys, which allowed to identify necessary adjustments to the programme and the infrastructure. Also, in the context of an inclusive, interdisciplinary and collaborative education approach (see Sections Value of a Collaborative Approach and Impact on Diversity and Equal Opportunities in Neuroscience Training), the EPO as coordinating layer proved essential, as it helped to facilitate collaborations and strengthen networks, drove progress, supported the provision and sharing of resources and ensured that all stakeholders have the necessary tools and resources to implement activities (Rincón-Gallardo & Fullan, 2016).

**Fig. 4:**
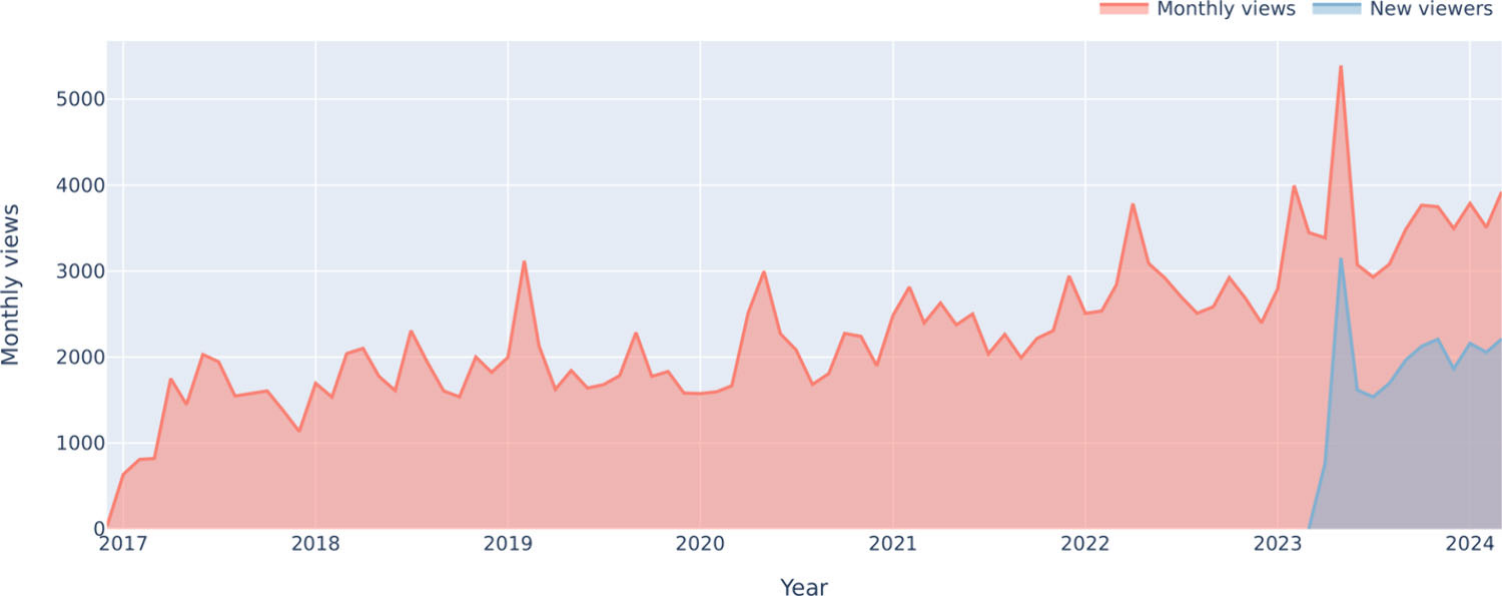
Monthly views (red) and proportion of new viewers (blue) on the HBP Education E-Library hosted on YouTube covering the period from December 2016 (first videos published) to March 2024. View numbers increased steadily along the HBP phases. Average monthly views from December 2016 to March 2020 (SGA1, SGA2): 1669.95 (SD: 500.70) and for April 2020 - March 2024 (SGA3 and beyond): 2833.65 (SD: 704.63)

**Fig. 5:**
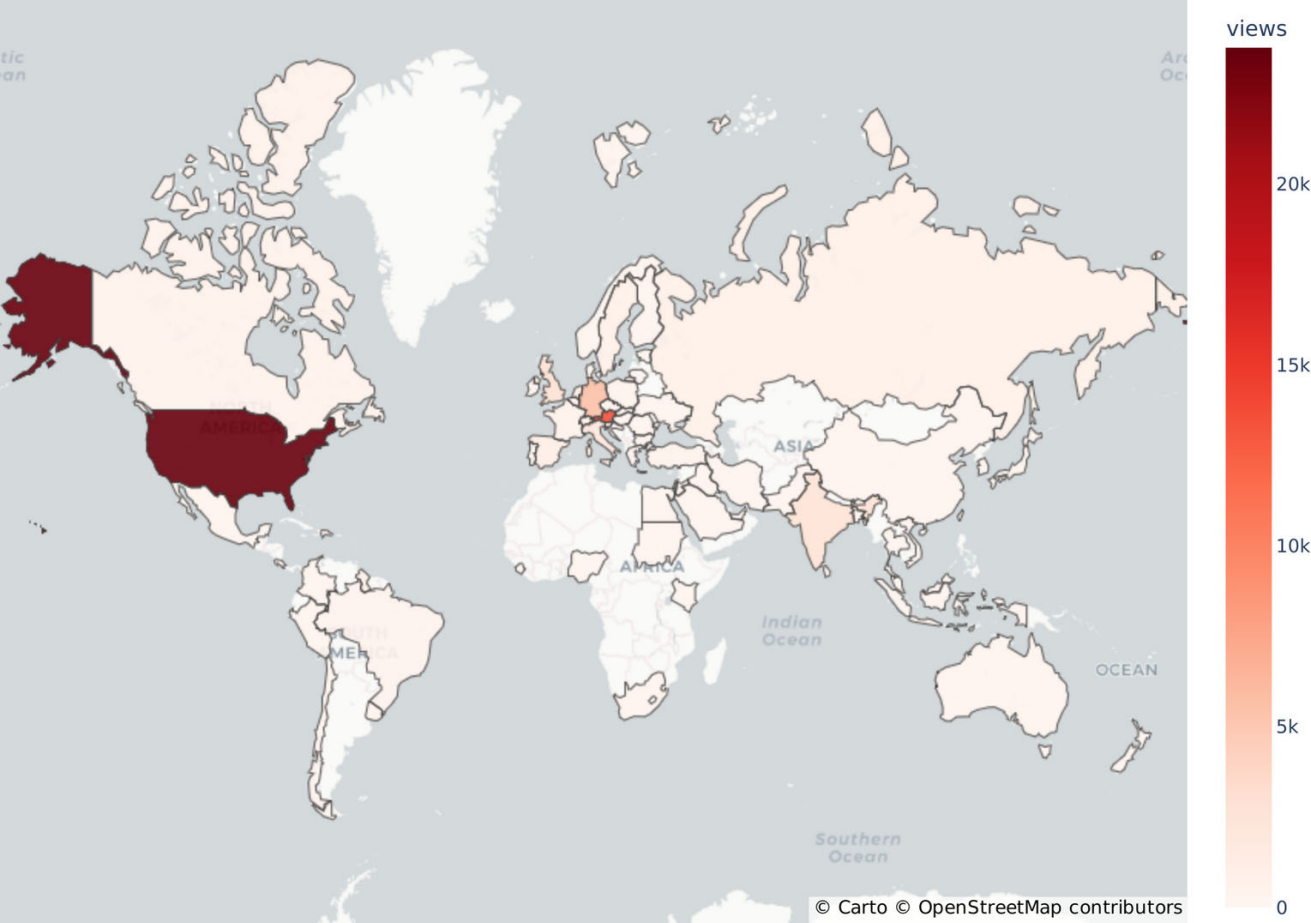
Number of views by country on the HBP Education E-Library hosted on YouTube covering the period from December 2016 to March 2024. E-Library videos have reached a wide geographic audience well beyond the European dimension of the HBP

### Adaptability of a Blended/Hybrid Educational Approach

While providing training on the digital infrastructure for brain research developed within the HBP, the HBP Education Programme also evolved to integrate digital tools into the training practice. The use of digital tools in education has increased over the past decades (Gubbins et al., 2023) in many disciplines, including neuroscience (Rajan & Pandit, 2022), with benefits for inclusivity and accessibility of training (an Viegen et al., 2021). The COVID-19 pandemic starting in early 2020 accelerated this trend and made online learning, and not just digital tools, common for many education programmes, from schools to universities and researcher training (Wee, 2022; Achakulvisut et al., 2020). Since the beginning, the HBP Education Programme has valued digital and online learning (Grasenick & Guerrero, 2020) along with in-person training, with a two-fold impact: making educational material available online during and after the events, and expanding the audience of training events (including those who could not afford to travel to an on-site event). Digital training contents (video material produced during the HBP Education Programme activities) contributes to the legacy of the HBP Education Programme: an e-library of courses and lectures, organised into corresponding topics following various HBP events, is shared with the public through the HBP Education Programme E-Library^4^ and made available on YouTube^5^.

**Fig. 6:**
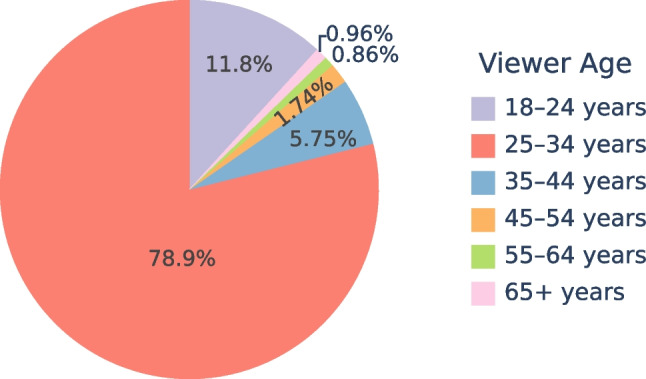
Number of views by age category on the HBP Education E-Library hosted on YouTube. E-Library videos have been mainly watched by young audiences with more than $90\%$ of views coming from individuals younger than 35

More than 700 videos are available in the E-Library, reaching close to 4, 000 monthly views by April 2024. Since March 2023, on average, $53.9\%$ of monthly views are from new viewers, as shown in Fig. 4. The views by countries indicate that the E-Library has reached geographic audiences well beyond the European dimension of the HBP, as shown in Fig. 5. For example, India and the United States of America are among the top five countries with the highest number of viewers. Regarding viewer age, Fig. 6 shows that the main age group using the E-library were 25-34 year-olds, followed by 18-24 year-olds, which indicates that the main target group of ECRs (including undergraduate and graduate students, OECD, 2019) is well reached by the E-Library content.

**Fig. 7:**
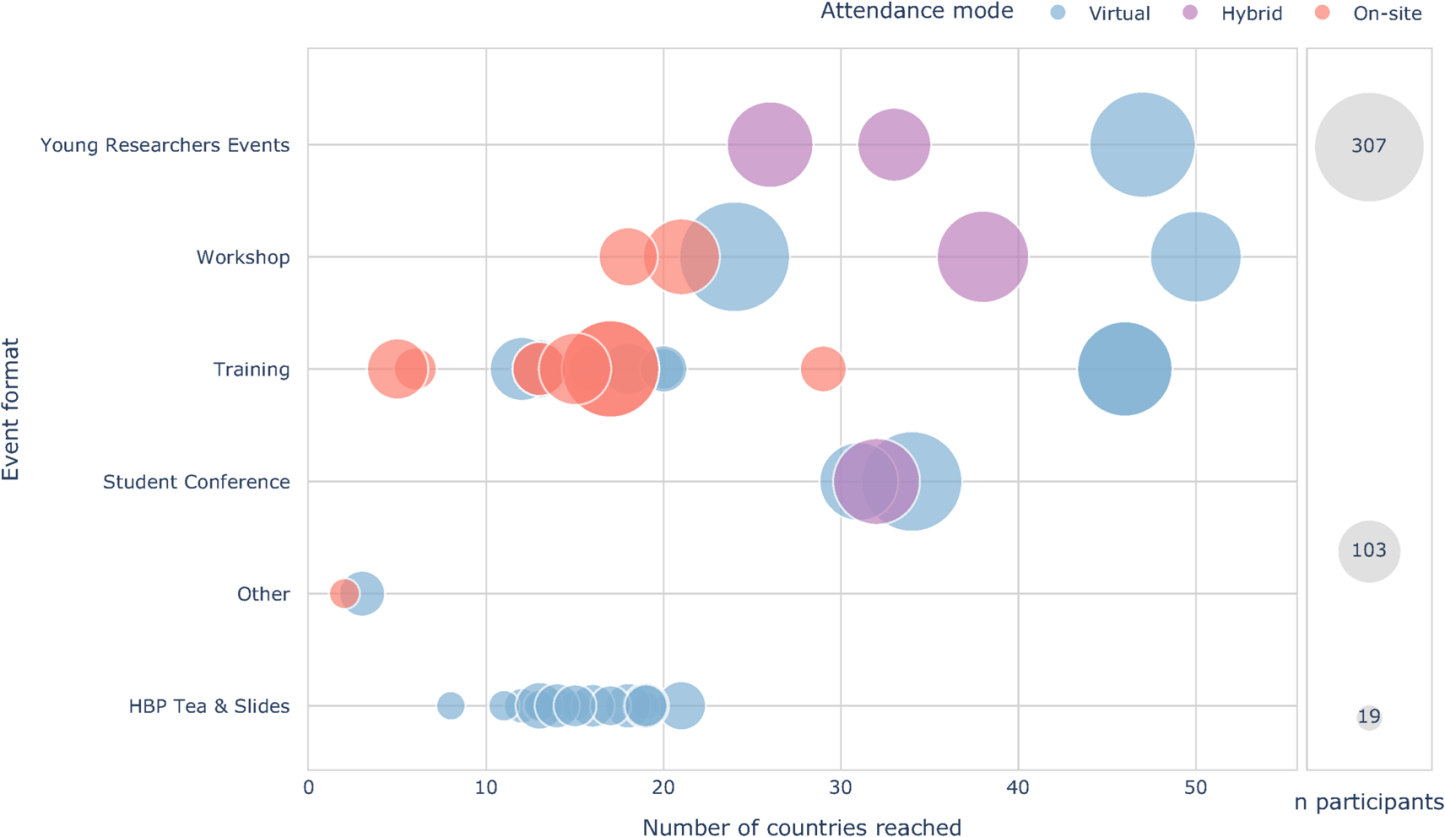
Worldwide reach of education events from 2020-2023. Bubble size represents the number of participants per event (the legend shows exemplary bubble sizes for the events based on median and min/max participants numbers). Hybrid and virtual events increased geographic reach of the HBP Education Programme. Also smaller formats, such as the HBP Tea & Slides webinars (see Section HBP Tea & Slides), reached between 8 and 21 countries per session, indicating increased geographic diversity of virtual formats. Geographic reach is defined as self-reported residence at registration

In addition to the digital education material, other online activities were implemented to enhance the cohesiveness of the community, help early-career researchers keep connected and engaged with the developments in the field, and provide an open space for interaction during times of physical disconnect of the pandemic years (2020-2022). The Tea & Slides events were launched in 2020 (see Section HBP Tea & Slides) and the Student Conferences and Young Researchers Events were moved to an online and hybrid format from 2021 onwards (see Sections HBP Student Conferences and HBP Young Researchers Events). As shown in (Fig. 7), having online and hybrid training events increased the number of participants and geographic reach, contributing to enhancing accessibility and equity of training across more countries. Particularly, formats that were moved from in-person to a hybrid/virtual format saw an increased geographic diversity. In general, also small-sized purely virtual events such as the Tea & Slides format (mean number of participants: 38.6) reached a considerable geographical diversity, with on average 15 countries represented per event.

The digitisation of content and hybrid format of events have ensured that educational material about the EBRAINS RI^6^ is available to researchers around the world. The variety of multimodal training and education material has been preserved and documented online^7^ to help ensure progressive adaptation opportunities by the research community to the developments of the HBP technical and scientific outputs.

## Value of a Collaborative Approach

The launch of large international initiatives for brain research (Amunts et al., 2016; National Institutes of Health, 2014; Yuste & Bargmann, 2017) and the availability of digital education platforms have facilitated the development of collaborative bottom-up approaches for neuroscience training. Within the HBP Education Programme, collaboration among ECRs, senior scientists, and other neuroscience training initiatives has been a core element of the educational offer. In the following section, we provide an overview of the diverse mechanisms implemented to increase collaboration inside and outside the HBP community through education.

### HBP Education Community

The involvement of students and young researchers in the HBP education activities was a substantial part of the Education Programme, with students playing a pivotal role, especially in shaping the format and content of education events. In particular, student representatives (SR) and ambassadors (SA) represented the interests of the student community within the HBP. They played a crucial role in strengthening the community by promoting awareness of the HBP Education Programme, and facilitating communication between students and members of the whole HBP community. Their main activities included (HBP, 2017a, b):

- Representing the student body during HBP Education Programme committee meetings and distribution of information within their respective network.
- Contributing to shaping the strategy for the HBP’s Education Programme in its operational phase by providing valuable input from a student perspective.
- Acting as a bridge between different groups within the HBP’s ecosystem by connecting the growing student community with the Education Programme Office and senior scientists, as well as fostering collaboration with the broader HBP consortium.
- Co-organising the annual HBP Student Conference with the EPO and voluntary students to facilitate networking opportunities and knowledge sharing among students involved in the programme.
- Organising networking events and student gatherings at the HBP Education Programme events, promoting a sense of community and fostering connections between participants.
- Organising student community contributions and side events at major HBP events like the annual “HBP Summits”, which were large conferences with hundreds of participants – an opportunity for students to get visibility for their work early on in their careers and to meet with their peers in the community.

Over the course of the three funding phases, lasting from 2016 - 2023 (SGA1-3), 14 men and 11 women participated as SA and SR. SRs were periodically elected by the ECR community for a voluntary duration.

To further develop and maintain the community, the HBP Education Programme Office maintained various communication channels to multiply the Programme’s reach, including a monthly HBP Education newsletter with 3, 800 subscribers, social media presence reaching more than 14, 000 followers across platforms by the end of the HBP, and an open HBP Student Community Slack Channel with more than 600 members.

**Fig. 8:**
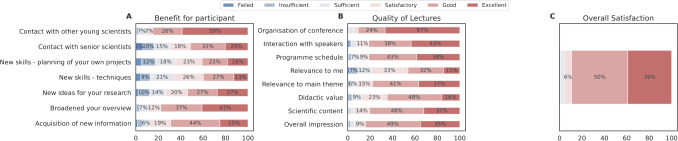
SGA1 and SGA2 HBP Student Conference survey results. Entries below $5\%$ are omitted

**Fig. 9:**
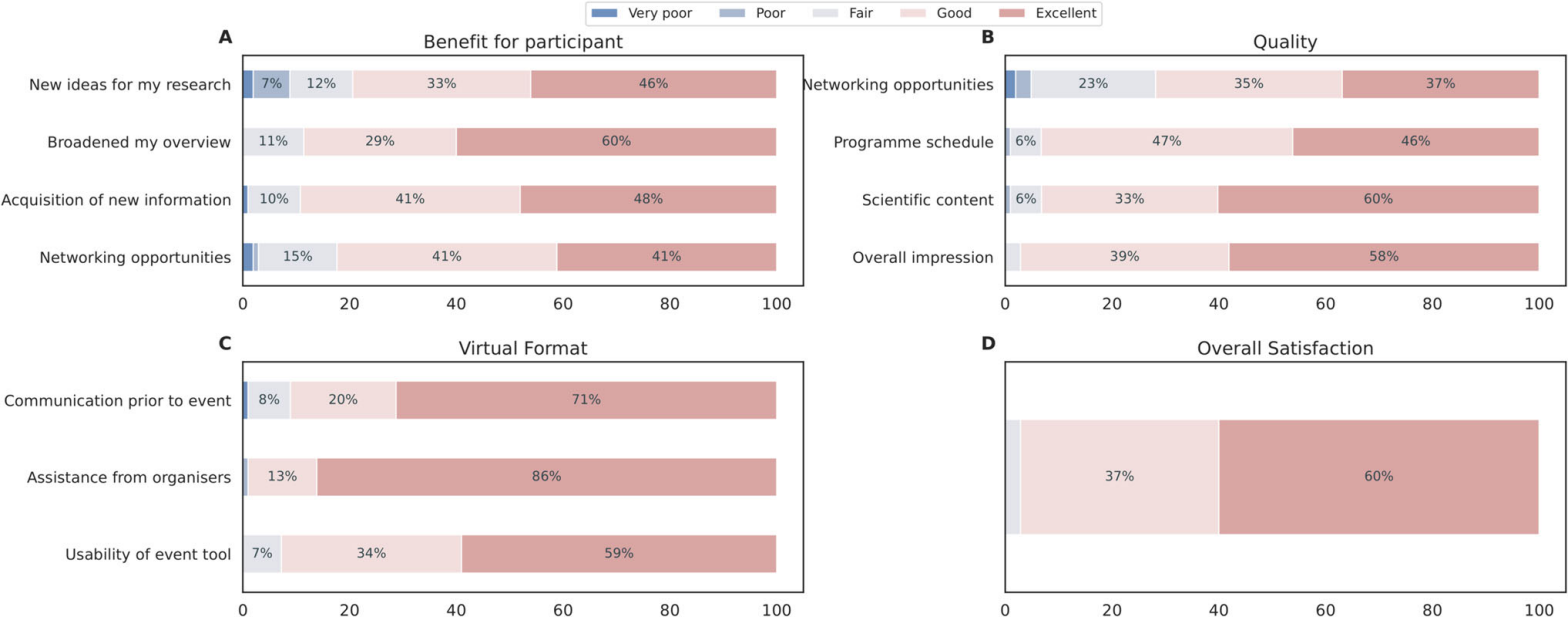
SGA3 HBP Student Conference survey results

### HBP Student Events

All student events, including the HBP Student Conferences, the Young Researchers Events and the Tea & Slides webinar series were organised by the SA for students inside and outside the HBP community. The scientific content was designed to encourage collaboration across disciplines (see Section Value of an Interdisciplinary Approach) and discussion with peers and principal investigators. Hands-on workshops and interactive sessions were organised for learning new skills and giving students new networking opportunities with other users and developers of tools.

#### HBP Student Conferences

Seven HBP Student Conferences (Simidjievski et al., 2017; Santuy et al., 2018; Urbain et al., 2019; HBP, 2020; Bogdan et al., 2021; Covelo et al., 2022; Cano-Astorga et al., 2023) were organised by ECRs for ECRs with the aim to encourage collaboration and scientific exchange across the fields of neuroscience, brain medicine and computer science. The idea was that student-organised conferences allow bottom-up perspectives and innovative ideas from the young scientific community, while providing a supportive environment for students presenting their work, often for the first time. ECRs had the chance to present their own research and engage in extensive discussions with peers and principal investigators from within and outside the HBP, often in an informal environment.

The event quality (scientific content, programme schedule and interactivity) and the organisational aspects (communication before the event, assistance from the organisers and usability of the event tool in case of virtual/hybrid events) were rated and assessed in post-event surveys. The mean outcome of the first four conferences is depicted in Fig. 8 (SGA1 – SGA2), and the 5th to 7th conference (SGA3) in Fig. 9. Between the 4th and 5th conferences, the survey format was changed to consolidate and simplify the questions, which reduced the number of questions and provided a streamlined polling process. In response to the COVID-19 pandemic, from the 5th conference onwards, a virtual or hybrid question format was implemented. The number of survey participation for the SGA1 and SGA2 phases and for SGA3 is further detailed in the Supplementary material, Section B.

Importantly, most respondents had a positive impression of the conferences (see *Overall Satisfaction* in both plots). According to the poll, many students may have used the conferences to learn more about other disciplines than their own (*Broadened your overview*). Based on the received feedback, student-led conferences represented a highly educational experience for ECRs gaining new knowledge (e.g., *New skills* and *Acquisition of new information* in Fig. 8, **A**) and know-how in highly rated lectures (e.g., *Scientific Content* in Fig. 9, **B**). Many students indicated that they gained many new ideas (see Fig. 9, **A**, *New ideas for my research*), particularly during the SGA3 phase. This may correlate with the increased networking possibilities provided. Furthermore, another aspect which may influence new ideas is that the students may appear more engaged when interacting with and between early-career researchers like themselves. An indication of this is reflected in *Contact with other young scientists*, in Fig. 9, **A**. The impact of the COVID-19 pandemic is visible in Fig. 9, B with a lower response rate for *Network opportunities*. This decline can be attributed to the online/virtual format, as networking was not as easily facilitated compared to in-person meetings (Karl et al., 2022; Standaert et al., 2023).

**Fig. 10:**
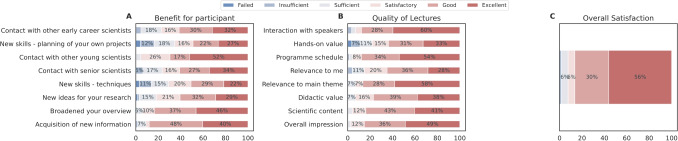
Young Researchers Events satisfaction survey results from the early SGA1 and SGA2 funding phase. Entries below $5\%$ are omitted

**Fig. 11:**
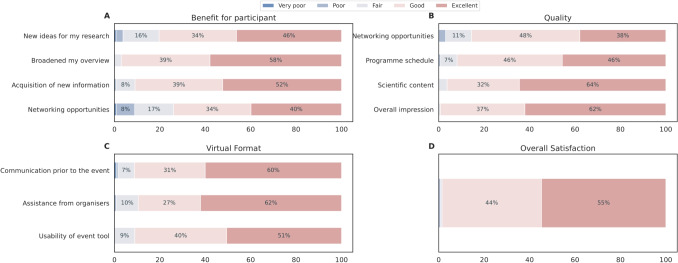
SGA3 Young Researchers Events satisfaction survey results present highly positive summary statistics during SGA3 that included virtual events. Overall satisfaction results improved

Overall, there is a noticeable rise in overall satisfaction across the conferences, as reflected by an increase in the response rate for the *Excellent* category for all questions (see Fig. 9). A potential source for this improved satisfaction may relate to increased experience and continuous consideration of feedback and suggestions from participants, as well as the expansion of the HBP research network, in terms of new scientists with a higher variety of backgrounds and experiences.

#### HBP Young Researchers Events

Seven free-of-charge events lasting from one to two days were organised collaboratively by senior researchers and ECRs for ECRs. These “Young Researchers Events” were held in person/hybrid in Budapest (Hungary), Geneva (Switzerland), Sofia (Bulgaria), Belgrade (Serbia), Copenhagen (Denmark), and Zadar (Croatia), while one was a virtual event.

Various scientific topics were addressed in interactive plenaries and hands-on workshops. The events offered ample networking formats for participants to connect with peers and experts, like the Science Bazaar, where event participants, including some senior researchers contributing to the event, were organised in groups discussing different topics. As displayed in Figs. 10 and 11, opportunities to meet with ECRs and speakers were highly valued (*Contact with other young scientists* together with *Contact with other early carrier scientists*). In contrast, contact to senior researchers was less well received (*Contact with senior scientists*). These satisfaction surveys indicate that, in line with the aims, these events helped broaden the perspective for almost all respondents (*Broadened my overview*) while providing only limited benefit to individual skill acquisition as per the feedback (see *New skills* in Fig. 10). This issue may stem from either the broad or specialised nature of the course contents. To mitigate this problem, it is essential to consider the diverse backgrounds of all participants, even before designing the course, which poses a considerable challenge (see also the lessons learnt box in Section Towards Integrated Neuroscience Education). While individual topics were rated more highly for SGA3, the overall satisfaction did not change between SGA1-2 and SGA3, especially in the *Excellent* class. With the possibility to join virtually in SGA3, the number of participants increased (see Supplementary material Section B). The fewer response categories and slightly different questions may influence the outcome as well. Additionally, *Overall Satisfaction* was an individual question, thus, participants may have based their answer on different factors rather than summing up their ratings of the individual categories. This could have resulted in the individual categories being rated more positively in the SGA3 period, without changing the overall satisfaction.

#### HBP Tea & Slides

During the pandemic, the HBP SA & SR, invited the science community to 16 virtual talks (HBP Tea & Slides) by HBP ECRs. Each session included two 10-minute talks followed by an interactive Q&A session with the audience. Speakers were encouraged to present their work to gain experience and to receive valuable feedback from their peers. The speakers could also request a certificate in order to add this experience to their CV. Particularly during the height of the COVID-19 pandemic in the years of 2020 and 2021, with restricted networking and travel opportunities, this format was a simple and effective method to enable ECRs to publicly present their work in an interactive and informal manner to an international and interdisciplinary audience, as indicated in Fig. 7.

### Set and Setting Strategies to Increase Engagement

One aspect of the programme that contributed to its success and is often overlooked is the organisational setting of the events. While formal education elements, such as lectures and poster sessions, and the choice of topics and lecturers are undoubtedly the focus of most programmes and conferences, the space and social context in which they take place is equally important, especially from the networking and collaboration perspective. One reason why these aspects are often overlooked could be that their success is predicated on their subtlety and passiveness. One cannot assume researchers will socialise. Poster sessions are often thought to achieve socialising opportunities, and indeed, they do invite people to interact with each other and discuss each other’s work to a certain degree. However, participants often tend to visit posters that are at least somewhat related to their studies while avoiding those that are far outside their own expertise. This creates a feedback loop where, for instance, physicists will tend to exclusively interact with other physicists, if poster sessions are the only means of socialisation. Approaches such as moderated introduction rounds often do not achieve the aim to familiarise participants and to lower the social barrier.

HBP Education Programme events created increased opportunities for networking across very different fields. Being set in comparably remote places like secluded mountain locations, such as Alpbach or Obergurgl in Austria, helped retain participants at the venue. Moreover, decreasing the attendance numbers deliberately increased the opportunities to socialise and form deeper connections. Barbecues, hikes and social evening can encourage communication in a very informal setting, fostering the building of relationships between the participants, both at a personal and professional level.

These factors, which seem at first glance to be inconsequential, must be given special attention in the planning phases of events. Creating quality opportunities to socialise is an important factor in building lasting collaborations (Aicardi, 2014), and should not be perceived merely as a means for increasing the enjoyment component (see Lesson 5 in the box in Section Towards Integrated Neuroscience Education).

### The HBP Mentoring Programme

To guide ECRs in their careers and potentiate the connection between them and senior researchers, the HBP offered a high-potential mentoring programme, developed by the Diversity and Equal Opportunities Committee. This was a mentoring partnership between an ECR and an experienced researcher (mentor), such as a group leader or someone already experienced in guiding students through their academic careers. One of the goals of this programme was to shape the mentee’s career goals with a clear and guided orientation, including the focus on successfully performing future tasks in an independent manner. The programme followed recommendations given by Colantuono (2012); Grasenick and Trattnig (2019), emphasising ways to mentor students based on inclusiveness while building self-confidence, self-reflection, and courage. Structured guidelines (Grasenick, 2017) helped the mentor-mentee pairs to formulate personalised goals that they worked towards over 6-8 weeks.

### Collaboration with Other Initiatives

The collaborative approach of the HBP Education Programme also aimed at creating and fostering bridges to other initiatives active in interdisciplinary research and education. From the outset, collaboration with international organisations, such as the Allen Institute for Brain Science and the International Neuroinformatics Coordinating Facility (INCF), was initiated for joint workshops, cross-promotion and knowledge exchange (Saria, 2018). These initiatives enabled the multiplication of the HBP Education offers as well as the tools and services offered by the EBRAINS Research Infrastructure. A train-the-trainer style Master Class Series co-organised with the International Brain Research Organisation (IBRO) gave the opportunity to senior scientists from Africa and the Asian-Pacific region to adopt EBRAINS tools for their own teaching and research^8^. As a result, collaboration with other initiatives facilitated the integration of new audiences, both geographically and disciplinary. The Young Researchers Event series contributed to fostering collaboration with researchers from countries not formally associated with the HBP (such as Lithuania, Croatia, Hungary, or Serbia) by co-organising the events with local universities or neuroscience associations and giving both HBP and local scientists space to present their research. Co-organised workshops with actors from associated fields, such as a collaborative workshop between the HBP and the European Academy of Neurology (EAN)^9^ helped to broaden the disciplinary audience. Collaborations were also useful as a standing exchange on strategies, methods, and ways to increase the impact of educative actions. HBP Education Programme members have been recruited as Observing Members at the INCF training and education subcommittee to provide input to INCF educational activities (Saria, 2018). Further, a collaboration with the International Brain Initiative (IBI) included participation of the HBP Education Programme representatives in the Training Task Force of the IBI Data Standards and Sharing Working Group, together with other IBI member organisations^10^. The main mission of the task force was to increase the impact of data generated by the international neuroscience community on science and health. This was achieved by ensuring that community members had access to training resources that promote data standards, facilitate data sharing, and provide guidance for obtaining and analysing these data sets. This mission was also multiplied during conferences, for example, at a SfN 2022 Satellite Symposium organised by the Training Task Force^11^.

### Interdisciplinary Collaboration for Responsible Research and Innovation Training

The major aims of the HBP Education Programme included a dedicated focus on Responsible Research and Innovation (RRI). This focus was in line with the overall approach of the HBP, which maintained a strong commitment to RRI and ethics. The initiative engaged a large team of social scientists and humanities researchers who collaborated closely with neuroscientists, computer scientists and engineers (Aicardi et al., 2020; Stahl et al., 2019; Ulnicane et al., 2023). A critical element of the RRI approach is aligning research with the needs and values of society. This alignment is facilitated through broad collaborations, bringing together a range of disciplines and stakeholders with an aim to anticipate, reflect, engage, and act on social aspects of research and innovation.

Members of the HBP RRI team worked jointly with the HBP Education Programme to ensure that topics of RRI and ethics were an integral part of the HBP education events. In addition to a dedicated three-day HBP Education workshop on RRI and ethics in 2018 in Stockholm, Sweden, training on social aspects was part of regular HBP Student Conferences and Young Researchers Events. Social science topics were also discussed at other events, such as during the HBP Tea & Slides webinar series. A unique feature of these RRI and ethics trainings in the HBP was that they were co-created within a long-term collaboration among HBP researchers from SSH (Social Sciences and Humanities) and STEM (Science, Technology, Engineering and Mathematics) disciplines as well as students, to make sure that the needs and questions of interdisciplinary brain research were addressed.

During the final years of the HBP, the RRI team developed an RRI capacity development programme offering training on topics such as data governance, dual use, neuroethics, and public engagement for HBP researchers, students, EBRAINS users and a wider audience (Ogoh et al., 2023). Some of these training elements, for example on dual use, was offered during the HBP Student Conferences and Young Researchers Events. The dual use workshops were developed in collaboration with the project-wide HBP Dual Use Working Group and regularly updated in line with students’ feedback (Ulnicane et al., 2023). These workshops introduced students to the key concepts of RRI, dual use of concern, and misuse and provided examples and ways to address dual use issues. The major emphasis during these interactive workshops was on student group discussions and plenaries, where students discussed how the issues of dual use and misuse might apply to their own research and how to address them. In this way, workshops encouraged reflection, mutual learning, and creation of collaborative networks of responsibility for thinking and dealing with social aspects of brain research.

## Impact Highlights

Thus far, we have described the HBP Education Programme and discussed the value of specific conceptual pillars that guided the evolution of education and training throughout the project. In this section, we will highlight the specific impacts of the programme on project execution, research and technology development, and the community.

### Interdisciplinary Skills

One of the challenges of interdisciplinary environments is addressing disciplinary pluralism, as most researchers are trained in specific fields. The HBP Education Programme aimed to create an interdisciplinary space by exposing participants to diverse disciplinary perspectives and approaches, both formally at conferences and workshops, and informally through personal interactions and conversations. These interactions help uncover knowledge gaps, challenge assumptions, and align terminology, thereby facilitating hypothesis formulation (Donovan et al., 2015). Such interdisciplinary exchanges increase awareness of the broader impact of the research performed by attending individuals and research groups, insights unlikely to be gained from mono-disciplinary conferences or events. Be it through the HBP education events and/or mentorship programmes, the HBP Education Programme contributed to build an interdisciplinary research environment, increasing networking opportunities and providing ECRs with a better understanding of the underlying scientific challenges of each discipline and how they relate to the project’s overarching goals, as seen in the “benefit for participant” question on the surveys like the one presented in Figure 9 (see also Supplementary material Section A.3 for a success story on this topic).

### Impact on Technology Development

Having an actively evolving Education Programme within the HBP also had an important impact on the development of the resulting research infrastructure and technology. First, the continued effort to provide reliable and flexible digital environments and tools for neuroscience training pushed the internal technology roadmaps to achieve the required levels of robustness and usability through the execution of the project (see Lesson 2 in the box 2 in Section Towards Integrated Neuroscience Education). Moreover, the feedback and requirements provided by students, who frequently became active end-users of the infrastructure, were critical for the co-design of the tools and services integrated into the EBRAINS RI (see also Supplementary material Section A.2).

In order to create a link between the needs of the end users and the technology development, the Scientific Liaison Unit (SLU)^12^ (see Glossary 1) was formalised during the last phase of the HBP. Its main goal was to foster a collaborative and integrated research environment. The SLU contributed to the EBRAINS research ecosystem by creating instructional materials, guides, and videos that facilitate data sharing and integration of research workflows. The EBRAINS SLU also contributed to educational activities and used them as platforms to interact with end users and gather requirements. An event of note, co-organised by the SLU and the Education Programme, was a tailored one-day workshop for the Austrian Neuroscience Association (ANA, 2022). In collaboration with the Education Programme, the SLU organised workshops and events to teach researchers, particularly those early in their careers, how to achieve their scientific goals using standardised workflows in the EBRAINS RI and provided mentoring and resources to help participants develop their ideas into structured requests for implementing their research. Díaz (2023) documents the results of one of such workshops.

The Education Programme together with the SLU fostered data sharing and the refinement of tools meant for collaborative digital research^13^. This is crucial for neuroinformatics (Eckersley et al., 2003) and had a direct impact in open science methodologies, enhancing the trust of scientists in the infrastructure and the community and increasing willingness to listen to new perspectives as well as new ways of thinking and solving problems. This increased collaboration, also reflected in a more homogeneous communication among participants in these events, allowed for fast and efficient interactions resulting in various open-access manuscripts, curated datasets and free licence software releases.

### Flipped Hierarchies

In most scientific disciplines, project ideas and directions are formed in a top-down hierarchy: initiated by the senior principal investigator, further passed down and refined by the more junior researchers like PostDocs and PhD students. The senior principal investigator would lead the conceptualisation of the idea, forming broader concepts that need to be investigated. At the same time, the junior researchers focus on the technical details related to the implementation and evaluation of the hypotheses. This approach is most often appropriate in an intra-disciplinary setting. However, it is less appropriate for larger collaborative interdisciplinary efforts such as the HBP. Here, ideas are often conceptualised jointly by investigators from different disciplines. In turn, each will then attempt to translate these concepts to their subordinates, often with incomplete information about the scope of the planned effort and potential technical difficulties. This is expected, as it is typically inherited from the complexity of interdisciplinary communication.

The various HBP student events, outlined in Section HBP Student Events, facilitated interactions and communications among ECRs, creating a community that cohesively contributed to scientific and training activities throughout the different phases and leaderships of the project (see Lesson 3 in the box 2 in Section Towards Integrated Neuroscience Education). Understanding and respecting disciplinary pluralism in this context was imperative. On the one hand, it allowed for communicating and understanding potential technical difficulties akin to the other disciplines but unforeseen or lost in the traditional top-down hierarchical communication. For instance, a researcher specialising in neuroscience has a much better understanding of the data (how it was measured and processed) than a researcher specialising in applied machine learning who has been tasked to analyse it, and vice versa. Such interactions allowed for removing many technical bottlenecks, which further accelerated the planned deliverables. On the other hand, these events enabled the seeding of new ideas, implicitly flipping the conventional top-down hierarchies. ECRs could jointly identify and conceptualise novel scientific questions and potential common research directions by sharing and presenting their work with other interdisciplinary peers. These ideas were still propagated upwards and further improved and contextualised by the respective senior investigators, but were more actionable as the technical challenges were more tangible (see Lesson 4 in the box 2 in Section Towards Integrated Neuroscience Education).

Bottom-up ideas created by early-career researchers and developed in collaboration with peers from other disciplines proved to help move their respective fields forward, while, on the other hand, it also had a positive effect on the careers (see success stories in Supplementary material Sections A.1, A.2 and A.3). Such a high level of interdisciplinarity was rarely seen in the past in the field of neuroscience and new research opportunities can arise from this interdisciplinary collaborative mindset. Therefore, these bottom-up approaches also bring ECRs in the position to push the boundaries of their fields early on in their careers.

### Impact on Diversity and Equal Opportunities in Neuroscience Training

As a European FET Flagship project (see Glossary 1), the HBP in general, and the Education Programme specifically, was strongly committed to enhance equal opportunities and gender equality. In this context, circumstances of early-career researchers, such as income and family obligations, culture, ethnicity and first language, were specifically considered of high relevance. In that effect, the EPO and SA joined the Diversity and Equal Opportunities Committee in the development of equal opportunities measures. The Gender Action Plan of the HBP was developed in a collaborative fashion (Grasenick, 2021), identifying “research and lectures” as a primary area for intervention. This approach ensured that both representation and content were in line with the European strategy for gender equality.^14^. The integration of gender dimensions into research content is not merely about increasing the representation of women in science, but also about enriching research agendas and methodologies with gender-sensitive approaches. Within the HBP Education Programme, guiding documents for researchers who were interested in offering a workshop included questions that requested to explain who would present the content, how relevant aspects of gender and diversity would be addressed in the research and how the diversity of the participants would be considered didactically. These guidelines, integrated and provided to the wider community via the EDI (“Equality, Diversity, and Inclusion) Toolkit^15^, were taken into account when evaluating proposals for training events. Workshops on how to consider gender and diversity in research, as well as on biases and career development, were offered as part of Student Conferences other education events. Students were encouraged to raise any specific needs or concerns related to participating in the workshops and lectures. One measure to make events more inclusive for a wide audience was to offer as many education events as possible free of charge. The focus on virtual events in the last phase of the HBP further helped contributed to this in line with a shift to digital offers during the COVID-19 pandemic. For on-site events, collaborations with HBP consortium members or other institutions, who offered on-site venues as an in-kind service, helped to achieve low-cost structures that did not require income from registration fees and lowered access barriers for low-income researchers. As a result, $71.4\%$ of events in the last phase of the HBP were accessible for free. For the remaining paid events, the Education Programme aimed to provide early-career researchers with financial support in the form of fee waivers or travel grants. These grants again considered diversity factors, by ranking grant applications not only on academic merit, but additionally according to the GDP per capita of applicants’ country of residence. Applicants from the underrepresented gender were given preference. The average proportion of women participating in education events increased from $39\%$ to $45\%$ throughout 10 years of the HBP, while the proportion of women speakers rose significantly from $16\%$ during the preparatory up to $39\%$ at the final phase of the project (Willis et al., 2023).

## Towards Integrated Neuroscience Education

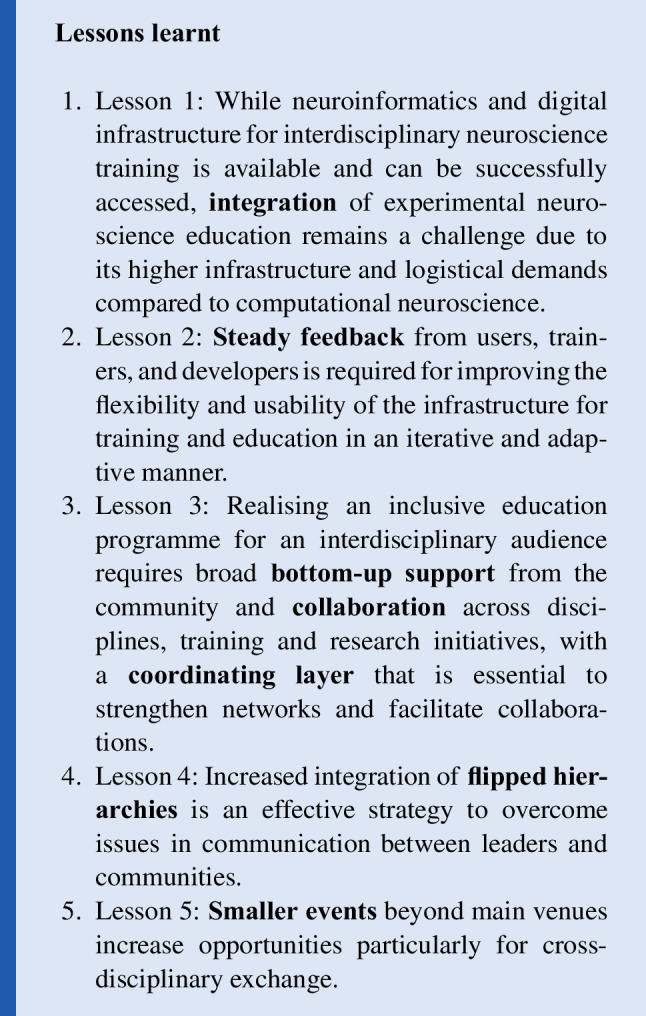



In the past decade, the integration of computer science and neuroscience has experienced a surge in popularity, impacting numerous disciplines (Marblestone et al., 2016; Jangid et al., 2020; Wang et al., 2020). However, keeping pace with this convergence presents a challenge for educational institutions, often hindered by rigid curricula, limited personnel and departmental silos. Smaller to medium-sized universities, in particular, frequently lack the interdisciplinary expertise and resources necessary, creating barriers that affect education, career paths, and scientific advancement.

Within the HBP Education Programme, efforts were undertaken to provide a platform that allows for an interdisciplinary education programme without borders by experts for everyone interested. Structured content presented by specialists, free and open, attracted a diverse community across European institutions. Impactful were an anti-hierarchical approach (flipped hierarchies), the mix of in-person and online offers, in formal and informal settings either driven by students or experts, with a strong focus on interdisciplinary skills – a format that in collaboration with other initiatives could help serve an even wider community. With the end of the HBP, elements of the HBP Education Programme were integrated into EBRAINS in a decentralised manner with a focus on the training and education around current EBRAINS tools and services provided by the EBRAINS ecosystem. Since the launch of the HBP Education Programme, several new international interdisciplinary education initiatives have emerged (Pradeep et al., 2022), alongside a surge in local digital offerings at universities, partly accelerated by the COVID-19 pandemic. These diverse offers should be better aligned and combined across institutions to leverage a wider potential for current neuroscience and computer science education efforts. A coordinating body overseeing educational efforts could ensure organisation, quality standards, and effective dissemination, fostering trust among educational institutions in the offerings. This would provide a more comprehensive and up-to-date education for interdisciplinary neuroscientists (Akil et al., 2016). In the European context, a decentralised neuroscience education platform within the existing European education ecosystem could complement and enhance traditional curricula at universities and higher research institutions. This approach would foster an ecosystem that harnesses local expertise across Europe, with ECRs playing a key role, and adapts and disseminates it to higher education institutions. For instance, universities could incorporate individual courses or structured programmes into their curricula digitally, saving resources while enhancing educational specialisation or interdisciplinarity. Simultaneously, decentralised workshops, hackathons, and practical courses on curated and shared datasets can complement existing courses to address at least partially the challenge of experimental training elements while still allowing small group learning opportunities and continuous feedback between users and developers of digital tools for neuroscience. Widespread, up-to-date interdisciplinary neuroscience training will remain a challenge, but a necessity to further improve by joint and coordinated action.

## Methods

This section provides a more in-depth examination of the methodology and data collection processes employed for the world cloud, survey results and E-Library statistics. Further details regarding the survey response rates and survey response acquisition are elucidated in the Supplimentary Material.

### HBP Student Conference Abstract Topics Word Cloud

To represent the topics of HBP student conferences, we clustered abstracts based on the predefined conference subtopics to which they were submitted, including (in alphabetical order): brain atlases; brain dynamics and disease modelling; brain simulation; brain simulation and brain-inspired architectures; cognitive and behavioural neuroscience; ethics and society; high performance computing; human brain organisation; medical informatics; medical informatics and clinical neuroscience; mouse brain organisation; neuroinformatics; neuromorphic computing; neurorobotics; organisation structure and function of neural systems; systems and cognitive neuroscience; theoretical neuroscience. A total of 395 abstract topics were included from 6 out of 7 HBP Student Conferences, for which this information was available. Clusters were then represented as a word cloud, using the online tool available at WordClouds (2024).

### Calculation of the Survey Results

Here we shortly explain the calculation of the mean survey results displayed in Sections HBP Student Conferences and HBP Young Researchers Events. The results are divided into three (SGA1-2) or four (SGA3) topics $T \!=\! \{\text {Benefit for participant},$ $\text {Quality}, \ldots \}$ and each topic has categories $C_t = \{\text {New ideas}$ $\text {for my research, Broadened my overview}, \ldots \}$ for a topic $t \in T$. For every category $c \in C_t$, the results are obtained as following:

$$\begin{aligned} \frac{1}{N}\sum _{n=1}^{N}v_{c} \, , \end{aligned}$$

where *N* is the number of events per SGA1-2 or SGA3 and $v_c$ are the votes (*Very poor*, ..., *Excellent*) per category *c*.

### Data Acquisition for HBP Education E-Library Statistics

The HBP Education E-Library is hosted as a public channel on YouTube. Anonymous data visualised in Figs. 4, 5 and 6 has been exported from the YouTube Analytics portal on 15 May 2024. The YouTube metrics used were (1) total views per month, (2) new viewers per month, (3) total views by country, and (4) total views by viewer age category. All metrics relate to the total number of videos available on the channel and cover the period from December 2016, when the first videos were published, to March 2024, except for the metric “new viewers per month”, which was only available by YouTube Analytics from April 2023.

While YouTube does not publish detailed methodology on their statistical algorithms, we work with the following assumptions regarding the calculation of metrics^16^: (1) Views are viewer-initiated intended plays of a video. (2) New viewers are unique viewers who discovered the channel for the first time by watching one of the channel’s videos, with unique viewers being defined as the estimated number of individual persons that watched content in a selected time period. (3) Viewer age is calculated based on views from persons who are logged-in to YouTube extrapolated to the full number of views using unpublished statistical inference methods. (4) Views by country are calculated using IP-address based location tracking, can however be limited by factors such as VPN and Proxies, mobile networks (due to dynamic allocation of IP addresses), and ISP (Internet Service Provider) factors. Due to potential inaccuracies and limited methodological insights into the YouTube Analytics data, the numbers presented cannot be seen as definite numbers. Additionally, it cannot be ensured that YouTube recorded metrics were consistent over the lifetime of the HBP Education E-Library channel, as their statistical algorithms may have evolved over time. Still, these limiting factors are assumed to cause only slight deviations from the definite numbers and therefore are highly unlikely to influence the overall statements presented in relation to Figs. 4, 5 and 6.

## Information Sharing Statement

The data used in this work, including the Python codes to create the plots of figures 3-11 in the main manuscript and figure 1 of the supplementary material, is available here: https://wiki.ebrains.eu/bin/view/Collabs/hbp-education-neuroinformatics/ and https://github.com/alperyeg/hbp_survey_plots.

## Supplementary Information

Below is the link to the electronic supplementary material.

Supplementary file 1 (pdf 133 KB)

## Data Availability

The original data that support the figures of this study are available from the corresponding authors, AG and JP, upon request and will be made available within a 14 day period.

## Footnotes

1 https://as-ai.org/

2 https://www.humanbrainproject.eu/en/education-training-career/education-programme/e-library/

3 Co-design projects were cross-cutting work activities within the HBP aimed at promoting exchange between scientific requirements and technology development, combining efforts across work packages (https://www.humanbrainproject.eu/en/about-hbp/project-structure/work-packages/).

4 https://www.humanbrainproject.eu/en/education-training-career/education-programme/e-library/

5 https://www.youtube.com/@HBPEducation

6 https://www.ebrains.eu/

7 https://www.humanbrainproject.eu/en/education-training-career/education-programme/e-library/

8 https://www.humanbrainproject.eu/en/education/virtual-masterclass-1/

9 https://www.humanbrainproject.eu/en/education/ebrains-workshops/medicaldata/

10 https://www.internationalbraininitiative.org/data-standards-and-sharing

11 https://brainminds-beyond.jp/satelliteopen@sfn_2022_ibi_data_WG.html

12 https://wiki.ebrains.eu/bin/view/Collabs/ebrains-scientific-liaison-unit-slu

13 https://drive.ebrains.eu/f/9f34fb0b132a485ebd2d/

14 https://commission.europa.eu/strategy-and-policy/policies/justice-and-fundamental-rights/gender-equality/gender-equality-strategy_en/

15 https://www.edi-toolkit.org/governance/#settheprinciplesforeventsandinteraction

16 https://support.google.com/youtube
