# Supplementary material for: Interdisciplinary and Collaborative Training in Neuroscience: Insights from the Human Brain Project Education Programme
Source: Neuroinformatics. 2024 Nov 6;22(4):657–78. doi: 10.1007/s12021-024-09682-6 (PMC11579076; doi:10.1007/s12021-024-09682-6)
Supplement: Supplementary file 1 — (pdf 133 KB) [file 12021_2024_9682_MOESM1_ESM.pdf]

A Success stories

A.1 Social robots for supporting post-traumatic stress disorder (PTSD) diagnosis and treatment

At the 3rd Human Brain Project (HBP) Curriculum workshop series on ”Modern trends in cognitive architectures and systems: From theory to implementation in natural and artificial agents,” held from 11-13 December 2019 at the University of Glasgow[1], the collaborative spirit encouraged by HBP Education Programme was exemplified. This event facilitated the initial meeting of Ziv Ben-Zion, a PhD student from Tel-Aviv University, and Guy Laban, a PhD student from the University of Glasgow. Despite their distinct research areas — social robots (Guy) and neurocognitive mechanisms of PTSD (Ziv) — their shared interests sparked a cross-disciplinary collaboration that extended beyond their individual projects.

This collaboration epitomises the HBP Education Programme's mission to promote multidisciplinary interactions that extend the frontiers of brain research and its applications. The HBP workshop provided the perfect milieu for the conception of an innovative project, focusing on the application of social robotics-based solutions in the diagnosis and treatment of PTSD. This interdisciplinary endeavour led to further discussions and meetings between Ziv, Guy and Emily S Cross (Guy's Principal Investigator), and culminated in the publication of a perspective article titled ”Social Robots for Supporting Post-traumatic Stress Disorder Diagnosis and Treatment” (Laban et al, 2022). The collaboration between Ziv and Guy continues to this day, testing the opportunity to use social robots to assist in the treatment of mental disorders. This sequence of events underscores the pivotal role that education programmes can play in fostering collaborative partnerships across fields with the development of new cutting-edge ideas. Without the unique convergence opportunity provided by the HBP event, such a fruitful collaboration might never have materialised, highlighting the programme's invaluable contribution to fostering innovative research synergies across disciplines.

A.2 Trainer knowledge gain from leveraging complex systems to non-experts

A collaborative endeavour materialised at the intersection of knowledge and practice through the organisation of two workshops held during the Life Sciences PhD meeting in Innsbruck[2] and the HBP Summit in Marseille in 2023[3]. These workshops provided an exposition of the EBRAINS environment and specific neuroscientific use-cases facilitated by the HBP Education Programme organised by Alper Yegenoglu and Michiel van der Vlag. The workshops served to introduce state-of-the-art high-performance computing (HPC) applications to scientists with limited or no prior experience in

[1] https://www.humanbrainproject.eu/en/education/participatecollaborate/curriculum/workshops/3rd-hbp-curriculum-workshop-series-cognitive-systems/
[2] https://biomed-phd.i-med.ac.at/life-science-phd-meeting/
[3] https://summit2023.humanbrainproject.eu/satellite-events-day/

working with supercomputers and compute clusters. The participants had to execute two different use-cases relating to research done at the Simulation and Data Lab Neuroscience at Forschungszentrum Jülich.

The first use-case includes a spiking neural network implemented in NEST (Gewaltig and Diesmann, 2007), which is optimised by L2L (Yegenoglu et al, 2022) to solve the mountain car problem, utilising the OpenAI Gym (Brockman et al, 2016) library. This task entails manoeuvring a virtual car, initially positioned at the base of a simulated valley, to ascend and reach a predefined target on the top of a hill. The second use-case features the optimisation of a TVB (Sanzleon et al, 2013) simulation, previously employed in a study concerning the integrated understanding of conscious and unconscious brain states and their underlying mechanisms (van der Vlag et al, 2023). Leveraging L2L, five model variables undergo automatic exploration to ascertain an optimal parameterisation for both synchronous and asynchronous brain states.

The knowledge acquired through the preparation and implementation of this material in a classroom setting surpasses the mere aggregation of its constituent parts. The preparatory phase yielded insights into the complexities inherent in establishing and operating a highly sophisticated infrastructure. A pivotal consideration for the tutors emerged: ensuring functionality not just for oneself, but for others as well. This experience engendered an implicit protocol emphasising comprehensive testing to guarantee system integrity. It facilitated a deeper understanding of the systems involved, particularly in the realm of user interactions, essential for successful deployment. The project went beyond simply running code; it required creating code that could discreetly install and configure hidden variables, invisible to participants.

A.3 Soft and hard skills for systems architects

The role of a system architect is sometimes misunderstood. In practice, it involves the production of both tangible outputs, such as documentation and design specifications, and intangible outputs, such as stakeholder alignment and conceptual frameworks. The goal is to balance system properties between internal and external requirements, ensure consistency across organisational and design boundaries, decompose complex systems into manageable subsystems, and integrate these components into a coherent whole, among other responsibilities (Muller, 2011). The profile of a system architect is often described as a generalist with areas of deep, specialised knowledge. Since architects are generalist positions with broad-reaching concerns, they contribute to the success of the system by collaborating with many teams.

The experience gained from organising and participating in cross-domain Young Researcher Events, HBP Summit open days, and participating in HBP Winter School of Neuroscience is invaluable, for example, in the semiconductor industry. Experience with the aforementioned events supported the creation of new neuromorphic platforms (such as Innatera's SNP (Inn, 2024)) and continued engagement within the neuromorphic research field (Yik et al, 2023).

The role of an architect in the semiconductor industry requires engagement with various stakeholders, internal and external. Stakeholders span different specialisations

and operate within different contexts (Muller, 2011), from business unit (e.g. marketing and sales representatives), direct customers, to internal experts across multiple domains (hardware design engineers, ML/AI engineers, software engineers, and platform engineers). The interdisciplinary experience gained from the HBP enabled the effective integration of diverse perspectives within meetings and facilitated bridging the understanding gap among specialists from different disciplines. This capability has been essential in promoting cross-functional collaboration and enhancing overall project outcomes.

B Survey response rates

**Event**	**Participants**	**Response**	**Phase**
1st HBP Student conference	79	55	SGA1
2nd HBP Student conference	67	40	SGA1
3rd HBP Student conference	42	28	SGA2
4th HBP Student conference	74	37	SGA2
5th HBP Student conference	215	46	SGA3
6th HBP Student conference	115	29	SGA3
7th HBP Student conference	150	30	SGA3
**Total**	**742**	**265**	

Table 1: Number of participants and response rate per HBP Student Conference.

**Event**	**Participants**	**Response**	**Phase**
HBP YRE 2016	64	18	SGA1
HBP YRE 2017	19	11	SGA1
HBP YRE 2019	42	15	SGA2
HBP YRE 2020	69	32	SGA2
HBP YRE 2021	227	78	SGA3
HBP YRE 2022	130	46	SGA3
HBP YRE 2022	117	13	SGA3
**Total**	**668**	**213**	

Table 2: Number of participants and response rate per Young Researchers Event.

Since the survey was on a voluntary basis we did not receive feedback from every respondent which explains the total response rate of 36% for the HBP Student Conferences (Table 1) and 32% for the YRE events (Table 2).

Following the end of the HBP Education Programme and to evaluate the challenges of interdisciplinary training from teachers' perspectives, a survey was sent to all speakers at HBP Education Programme events in March 2024, through SurveyMonkey. We received answers from 38 speakers, including 19 principal investigators, 9 postdoctoral researchers and 10 researchers.

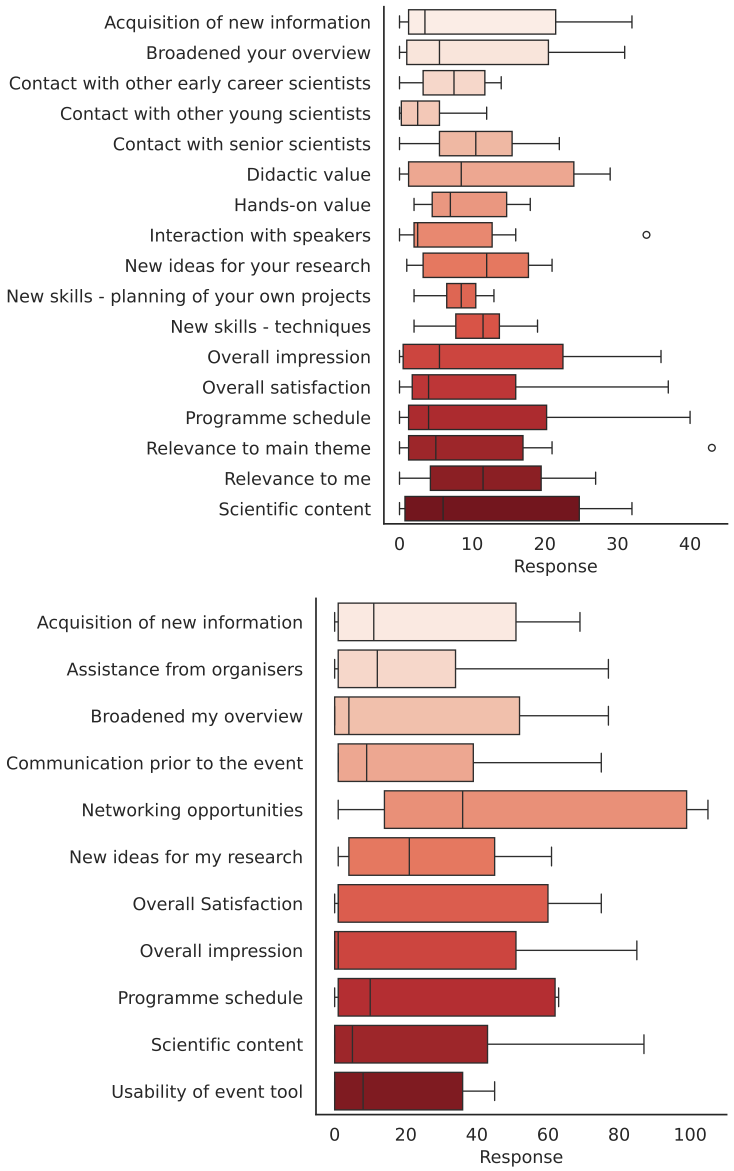

Fig. 1: Box plots displaying the survey participants' cumulative response per category. Each box displays the first to the third quartile. The horizontal bar displays the median, the whiskers depict the highest and lowest response rate. The circles indicate outliers. Top SGA1 and SGA2 phase. Bottom SGA3. The data is the same as in Figure 10 and 11 respective.

Survey results from the YRE events are visualised in Figure 1, illustrating the cumulative responses for each category $c \in C$, with $C =$

{Acquisition of new information, . . .}:

$$\sum_{n=1}^{N} v_c \,,$$

where v_c are the votes (*Very poor*, ..., *Excellent*) per category c and N is the number of events per SGA1-2 or SGA3 phase. With the option to participate online in SGA3, an increase in response rate compared to SGA1 and SGA2 can be observed. Same topics indicate a similar response rate (e.g. *Acquisition of new information*), often accompanied by a low median suggesting that most responses were clustered at the positive end of the scale.

The Python codes to create the plots of Figures 3, 8-11 of the main manuscript and for Figure 1 of the annex are available at GitHub (https://github.com/alperyeg/hbp_survey_plots).

References

(2024) Innatera's Spiking Neural Processor (SNP). URL www.innatera.com/resources

Brockman G, Cheung V, Pettersson L, et al (2016) Openai gym. arXiv preprint arXiv:160601540

Gewaltig MO, Diesmann M (2007) Nest (neural simulation tool). Scholarpedia 2(4):1430. https://doi.org/10.4249/scholarpedia.1430

Laban G, Ben-Zion Z, Cross ES (2022) Social robots for supporting post-traumatic stress disorder diagnosis and treatment. Frontiers in psychiatry 12:752874. https://doi.org/10.3389/fpsyt.2021.752874, URL https://doi.org/10.3389/fpsyt.2021.752874

Muller G (2011) Systems architecting: A business perspective. In: INCOSE International Symposium, Wiley Online Library, pp 1845–2142

Sanzleon P, Knock SA, Woodman MM, et al (2013) The virtual brain: A simulator of primate brain network dynamics. Frontiers in Neuroinformatics 7(MAY). https://doi.org/10.3389/fninf.2013.00010

van der Vlag M, Kusch L, Destexhe A, et al (2023) Vast tvb parameter space exploration: A modular framework for accelerating the multi-scale simulation of human brain dynamics. 2311.13337

Yegenoglu A, Subramoney A, Hater T, et al (2022) Exploring parameter and hyper-parameter spaces of neuroscience models on high performance computers with learning to learn. Frontiers in Computational Neuroscience 16. https://doi.org/10.3389/fncom.2022.885207, URL https://www.frontiersin.org/articles/10.3389/fncom.2022.885207

Yik J, Ahmed S, Ahmed Z, et al (2023) Neurobench: Advancing neuromorphic computing through collaborative, fair and representative benchmarking. arXiv 2023. https://doi.org/10.48550/arXiv.2304.04640
